# Long-term incorporation of manure with chemical fertilizers reduced total nitrogen loss in rain-fed cropping systems

**DOI:** 10.1038/srep33611

**Published:** 2016-09-21

**Authors:** Yinghua Duan, Minggang Xu, Suduan Gao, Hua Liu, Shaomin Huang, Boren Wang

**Affiliations:** 1 National Engineering Laboratory for Improving Quality of Arable Land, Institute of Agricultural Resources and Regional Planning, Chinese Academy of Agricultural Sciences, Beijing 100081, China; 2 USDA Agricultural Research Service, San Joaquin Valley Agricultural Sciences Center, Parlier, CA, 93648-9757, USA; 3 Institute of Soil and Fertilizer, Xinjiang Academy of Agricultural Sciences, Urumqi 830091, China; 4 Institute of Plant Nutrition and Agricultural Resources, Henan Academy of Agricultural Sciences, Zhengzhou, Henan 450002, China

## Abstract

Improving soil fertility/productivity and reducing environmental impact of nitrogen (N) fertilization are essential for sustainable agriculture. Quantifying the contribution of various fertilization regimes to soil N storage and loss has been lacking in a wide range of spatiotemporal scales. Based on data collected from field experiments at three typical agricultural zones in China, soil N dynamics and N changes in soil profile (0–100 cm) were examined during 1990–2009 under chemical fertilization, manure incorporation with fertilizer, and fertilizer with straw return treatments. We employed a mass balance approach to estimate the N loss to the environment after taking into account soil N change. Results showed a significant increase in soil N storage under manure incorporation treatments, accompanied with the lowest N loss (ave.20–24% of total N input) compared to all other treatments (ave.35–63%). Both soil N distribution and mass balance data suggested higher leaching risk from chemical fertilization in acidic soil of southern China with higher precipitation than the other two sites. This research concludes that manure incorporation with chemical fertilizer not only can achieve high N use efficiency and improve soil fertility, but also leads to the lowest total N loss or damage to the environment.

Use of nitrogen (N) fertilizer has been increasing continuously worldwide in the last few decades, especially in countries with rapid economic growth such as China, India, and Brazil^1^,^2^. As a result, crop yields have increased steadily and food security has been improved. However, since the 1990 s, over-fertilization has resulted in serious environmental degradation including eutrophication of surface waters, nitrate pollution of groundwater, acid rain, soil acidification, greenhouse gas emissions, and air pollution^2^,^3^. Thus, we face a continuous challenge in the use of chemical fertilizers and it is essential to develop the optimum nutrient management strategies for sustainable agriculture.

High crop yield almost always requires the application of more N to the soil than is removed by the crop^4^, which means inevitable N loss or environmental consequences from N fertilization. The best management practices (BMPs) target the lowest environmental impact, which is challenged by the desire of achieving the maximum yield. Some studies have shown that high yield could be achieved with reduced N fertilizer applications^5^,^6^. However, over a long period of time, depletion of soil N would occur and decreased soil N storage would reduce soil productivity over time^7^. Therefore, both plant uptake (crop yield) and soil N replenishment should be used to evaluate fertilizer management strategies.

Determining N loss from a cropping system can assist in evaluation of both N use efficiency (NUE) and environmental impact. In this paper, we refer to the N loss as the total loss to the environment that may include runoff, leaching (chiefly as nitrate, NO_3_^−^), and gas volatilization or emissions (e.g., NH_3_, N_2_O, N_2_, etc.). The concept of N balance (N input - N output by crop harvest removal) has been used to estimate the risk of N loss associated with specific farming practices at the field scale^8^,^9^. However, this approach does not take into account the changes in soil N storage, i.e., soil fertility or productivity, and there are practical difficulties in determining soil N status change in short-term field experiments. In this regard, long-term field experiments provide an advantage as they can reveal the cumulative effects of management practices on soil N storage and environmental impact^10^. In addition, changes in N distribution in the soil profile can indicate leaching risks after long-term fertilization because N in surface soil is available for plant uptake and downward movement signals leaching potential.

To develop efficient nutrient management practices in several important agricultural regions in China, long-term fertilization experiments were established in 1990 with one of the popular dry-land cropping systems, wheat and corn rotation. Previous reports have concluded that adequate phosphorus supply for wheat and manure application for corn are both efficient methods for improving yield and the overall NUE^11^,^12^. These studies, however, did not examine N distribution in the soil profile nor did they quantify the contribution of N fertilizer source to soil N replenishment or loss. The specific objectives of this paper were to: 1) examine the N distribution changes in soil profiles; 2) evaluate the fate of N in the pathway of soil accumulation, crop uptake, and total environment loss using a mass balance approach after 19 years of different field fertilization regimes.

## Results

### Changes in total N content in surface soil (0–25 cm depth)

**Figure 1:**
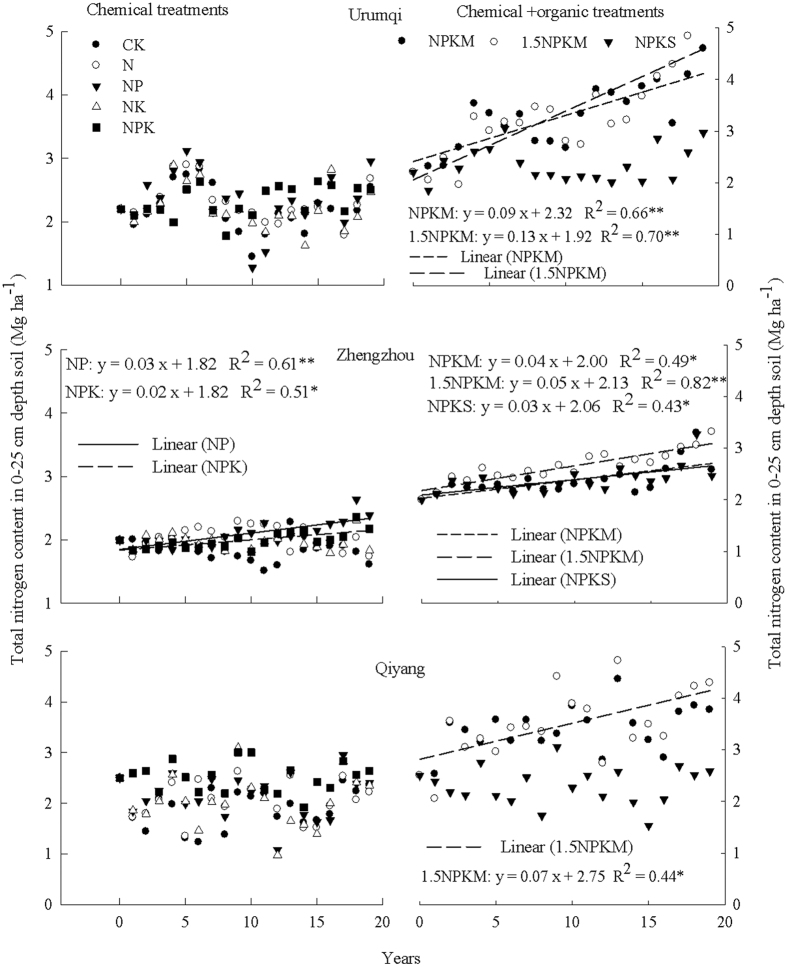
Changes of total nitrogen content at a 0–25 cm soil depth from various fertilization treatments over 19 years at Urumqi, Zhengzhou and Qiyang. Only significantly increased or decreased changes were shown with the linear regressions in the figure. **Significant at the 0.01 probability level, *Significant at the 0.05 probability level. CK, no fertilizer; N, chemical N fertilizer; NP, chemical N and P fertilizers; NK, chemical N and K fertilizers; NPK, chemical N, P and K fertilizers; NPKM, chemical NPK combinations with manure; 1.5NPKM, 1.5 application rate of NPKM; NPKS, NPK with corn stover or wheat straw returned.

The data were collected from a long-term experiment (1990–2009) at three sites: Urumqi (northwest), Zhengzhou (central), and Qiyang (south) of China. The eight treatments tested were the same at each individual site including: non-fertilization (CK); chemical N (N); N and P (NP), N and K (NK), and N, P, and K (NPK); chemical NPK combinations with manure (NPKM); 1.5 application rate of NPKM (1.5NPKM); and NPK with corn stover or wheat straw returned (NPKS). Overall, there was more N accumulated in soil for the manure treatments (NPKM and 1.5NPKM) than for the urea treatments (Fig. 1). Linear regression analyses indicated that the 1.5NPKM treatment had resulted in a significant increase in the soil N content at 0.13, 0.05 and 0.07 Mg ha^−1^yr^−1^ at the Urumqi, Zhengzhou and Qiyang, respectively. A significant increase in soil N content was also determined in the NPKM treatment at Urumqi (0.09 Mg ha^−1^yr^−1^) and Zhengzhou (0.04 Mg ha^−1^yr^−1^), but not at Qiyang. Soil N content had no significant changes during the 19 years for the chemical treatments at Urumqi and Qiyang. At Zhengzhou, the N content increased significantly for all N plus P treatments (NP, NPK and NPKS).

### Changes in total N content in top 100 cm soil after 19 years of fertilization

The results of variance analysis on soil N content in the top 100 cm from the various fertilization treatments, changes with depth, and their interactions across all three study sites are shown in Table 1. Soil N content varied significantly between the treatments and with depth, but no interaction was observed between the treatment and depth. Further tests on the differences in the means among the treatments and depths are given in Table 2. Based on the data from all three study sites, the soil N content for 1.5NPKM treatment (10.2 Mg ha^−1^) was significantly higher than all other treatments (7.3–9.1 Mg ha^−1^). A significant difference was also determined between NPKM (9.1 Mg ha^−1^) and CK (7.3 Mg ha^−1^). There were no significant differences in the N content among all the chemical fertilizer treatments nor from the CK. Regarding soil depth, the N content in the 0–20 cm soil was significantly (30%) higher than in the 20–40 cm soil and the N contents in both layers were significantly (43–100%) higher than those below 40 cm soil depth. There was no significant difference in the N content for soil depths deeper than 40 cm. Although the data indicate a significant increase in N storage in the soil, there were apparent differences among the study sites.

**Figure 2:**
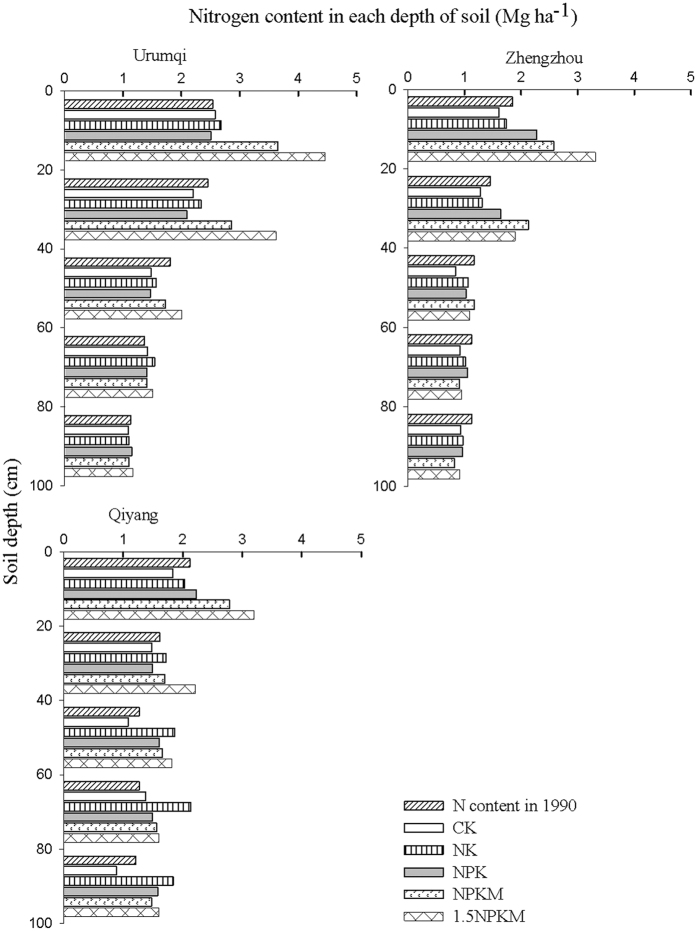
Total nitrogen content in soil profiles at the beginning (1990) and after 19 years of fertilization treatments (2009) at Urumqi, Zhengzhou and Qiyang. To increase the legibility of the figure, only the N contents for CK, NK, NPK, NPKM and 1.5NPKM are shown because of the similarity between N and NK, and between NP, NPK and NPKS. The abbreviations of fertilization treatments are the same as presented in Fig. 1.

Figure 2 shows the total N content in the top 100 cm soil profile sampled after 19 years of fertilization treatments. Differences between treatments were observed mainly in the top 40 cm soil. The soil N content was 37–46% higher for NPKM than for NPK at the 0–40 cm soil depth at Urumqi. At Zhengzhou, the N contents in 0–40 cm soil depth ranged from 1.9–3.3 Mg ha^−1^ for the NPKM and 1.5NPKM treatments, and 1.3–2.3 Mg ha^−1^ for NK and NPK treatments. However, at Qiyang the N contents at the 60–100 cm soil depth were higher for the NK treatment than for manure treatments whereas the opposite was true at the 0–40 cm soil depth. Compared with NPK and NPKM treatments, the NK fertilization resulted in 34–37% and 15–25% higher N contents at the 60–80 cm and 80–100 cm depths, respectively. The data indicate significant N downward movement from the chemical N treatments at Qiyang, i.e. potentially high leaching loss.

Compared with initial N content before the field experiment in 1990, the manure treatments resulted in a higher N content for all three sites by 2009. The chemical fertilization treatments resulted in a similar N content at the 0–40 cm soil depth for all three sites after 19 years of fertilization. Compared to 1990 values, the N content in 2009 was similar at Urumqi, lower at Zhengzhou, and higher at Qiyang in soil deeper than 40 cm. Using the N content data in 1990 and 2009, the annual changes in the top 100 cm soil N was calculated and given in Table 3. Both increase (positive numbers from manure treatments) and decrease (negative values from unfertilized or unbalanced fertilization treatments) in soil N storage were observed.

### Estimates of total N loss to the environment from different fertilization treatments

The annual N losses ranged from 52–205, 64–274, and 75–160 kg ha^−1^ yr^−1^ at Urumqi, Zhengzhou and Qiyang, respectively (Table 3). The lowest N loss was from the NPKM treatments (52–75 kg ha^−1^ yr^−1^), and the highest N loss was from the N or NK treatments (up to 274 kg ha^−1^ yr^−1^) at all three sites. The N loss percentage was 29–59%, 3–53%, and 14–27% lower from the NPKM treatment than from the chemical treatments at Urumqi, Zhengzhou and Qiyang, respectively. Averaging the three sites, 20–24% of applied N was lost for NPKM and 1.5NPKM while more than 35% was lost for chemical fertilization treatments.

Table 3 also presents the fate of N after the 19 years of fertilization treatments in terms of plant uptake (data from Duan *et al*.^11^) and changes in soil N that were used to compute the total N loss to the environment. The highest NUE was observed in NPKM treatment both at Urumqi and Qiyang (54% and 49%, respectively), and the NUE was similar among NP, NPK, NPKM and NPKS treatments at Zhengzhou (70–76%). The N and NK treatments had the lowest NUE (14–39%) in all three sites. For soil N, the highest increase in soil N was in manure applied treatments (22–29% increase) while others were all below 16%, average of the three sites.

## Discussion

### Estimates of total N loss

Korsaeth and Eltun^13^ concluded that average N balance predicted 86% of the variation in N loss. The disparity was due to the failure to include soil N storage in N mass balance. Using N mass balance approach and taking into account the soil N storage changes has allowed the more accurate estimates of total N loss to the environment in a crop production system. The data from this research have shown that soil N storage change indicating soil fertility/productivity can be different significantly for fertilization treatments (Table 3). Soil N storage varied from 14% depletion to 43% increase among the fertilization treatments that would have a direct impact on the estimate of environmental N loss. Thus, the mass balance after considering soil N change allowed more accurate estimates of the total N loss among the treatments (Table 3).

The estimated total N loss was also linked directly to NUE. Our study has shown that up to 57% of N fertilizer is accounted for in plant uptake and storage in soil (Table 3), which is close to those rates (50–65%) observed in Europe and USA^14^, and higher than that reported in China (30%, without considering the soil NUE)^15^. Thus, taking into account the soil N storage changes are important for evaluating N loss and developing sound management practices.

### Long-term manure incorporation reduced N loss

Our results showed that the average total N loss to the environment from the manure incorporation treatments (NPKM or 1.5NPKM) was 20–24% of total N applied while it was 43% from the commonly used balanced NPK fertilization treatment (Table 3). The data suggest that manure in combination with chemical fertilizer can be an effective management strategy to reduce N pollution to the environment. Although there were no apparent differences in plant uptake, soil N from the NPKM treatment increased ~7 times or higher significantly than the NPK treatment. The 1.5NPKM treatment resulted in relatively higher N loss than NPKM, most likely due to the unnecessarily higher total N amount applied because a lower NUE for the 1.5NPKM and similar grain yield were observed^12^.

Providing a steadily available N source by building up of soil organic matter must have contributed to the positive outcome of manure application^15^. Another important aspect was the enhanced microorganism activity in the soil^16^. Long-term application of NPK with manure was found to increase soil microbial biomass, which increased the amount of N immobilized by biotic process and reduced losses accordingly^17^,^18^. Although many studies indicate manure application could increase the risk of N leaching, our study demonstrated that a combination of manure and chemical fertilizer as 70% and 30% N resulted in not only the lowest N loss, but also with either higher or similar NUEs than those from 100% chemical fertilizer N (Table 3). The results showed that 70% replacement of chemical fertilizer by manure provided sufficient N for plant needs during the growing season, which must have resulted from mineralization of manure over time. We assume that the proper ratio of this combination would vary a little depending on fertilizer sources (N content), soil properties, and plant characteristics. The results imply that when applied properly, combination of manure with chemical fertilizer is an effective management strategy in agricultural production to significantly improve soil fertility (indicated by N storage increase), increase NUE or yield, and minimize N loss in the rain-fed annual cropping system in both alkaline (Urumqi and Zhengzhou) and acidic (Qiyang) soils.

Early analysis showed that the straw return did not significantly increase yield of corn and wheat in the double-cropping systems in the long-term experiments^12^. The results from this research (Table 2) showed that there was no significant difference in soil profile N between NPKS and NPK treatments. The straw return had generally higher N loss than the manure applications with exception for the 1.5NPKM at Zhengzhou (Table 3). Zhang *et al*.^19^ found that manure and straw have contrasting effects on organic N mineralization pathways due to different chemical, structural, and thermal properties. Mao *et al*.^20^ reported that manures are depleted in aromatic ring compounds and enriched in nonpolar alkyl compounds of the calcium humate fraction, which might enhance peptide contributions and thus improve soil N supply. In contrast, the compounds in plant residue are mainly recalcitrant carbon (celluloses, hemicelluloses and lignin)^21^,^22^. The C/N ratio of organic materials is another important factor that regulates N mineralization. From long-term application, soil receiving repeated applications of manure (low C/N ratio) and straw (high C/N ratio) were found to result in different microbial communities^23^,^24^, leading to different mineralization and immobilization rates of N in soil^25^. However, our knowledge on the soil N cycling related microorganism responses and regulation is still limited. Future research should strengthen the understanding on the mechanisms, especially the response of N transformation microorganisms to application of manure and crop residue that are associated with N availability to plants.

### Nitrogen movement and other losses

The N distribution in soil profile among the fertilization treatments appeared to follow the same pattern with relatively lower content in deep depths at Urumqi and Zhengzhou. At Qiyang, however, the N content below 60 cm depth especially in the N (not shown) and NK treatments was higher than all other treatments implying a higher N leaching risk (Fig. 2). Qiyang is located in the south of China with a high precipitation rate and an acidic soil (pH 5.7) due to high weathering and leaching potential. The higher precipitation at Qiyang (Table 4) would indicate higher leaching risks compared to the other two sites with lower rainfall. To more accurately assess the leaching loss, volatilization loss such as ammonium needs to be made, which is our on-going research.

Figure 2 shows that although Qiyang had the highest N leaching risks as shown by the similar N amount throughout the profile, the two alkaline soils also showed that at 100 cm soil depth the N amount was about half of that in surface soil for most of the treatments. The similarity between all fertilized treatments to the control may indicate historical leaching before the long-term experiment established. The data imply that the total N applied in this study could be further reduced and/or applied in a changed fertilization schedule following plant growth curve that will lead to even higher NUE than those reported in Table 3. At Qiyang, it is particularly more important to modify the fertilization schedule by reducing the initial amount of fertilizer applied and by applying the fertilizers several times during the growing season to meet plant needs. Multiple fertilizer applications have not been popularly adopted or enforced in practice, but is an efficient way to further improve NUE according to the data. Meisinger and Delgado^26^ summarized that the universal tools for managing N leaching include understanding the soil-crop-hydrologic cycle, avoiding excess N applications, and applying N in phase with crop demand. The data from this research indicate more feasible options should be pursued to develop the best management practices for N resource in the rain-fed cropping systems.

The estimated total N loss to the environment varied from average 20% for the NPKM treatment to 63% for the N treatment from this long-term study. The loss included mainly potential losses from leaching and gases through ammonia (NH_3_) volatilization and other forms of gases including greenhouse gas (N_2_O). Ju *et al*.^27^ studied N loss pathways in the North China Plain with wheat/corn double cropping system and chemical fertilizer application range of 263–325 kg ha^−1^. NH_3_ volatilization accounted for 19–24% and leaching loss was up to 12% with plant uptake of 26–46%. By taking the NH_3_ loss into account, the N loss would be in the range of 16–38% for all the chemical fertilization treatments including the NPKS but little leaching from the NPKM treatments based on the data in Table 3. Our study results indicate that the total N loss can be reduced to ave. 20% or lower by simply incorporating manure in the fertilization regime that reduced the loss more than 50% from chemical fertilizations (Table 3). These data strongly support that combination of chemical and manure fertilizers can provide the solution to reduce the environmental impact from chemical N fertilizer use. These data can apply directly to rain-fed systems similar to the studied climate conditions but may also apply to irrigated agriculture.

In summary, based on long-term field experiments and using the mass balance approach, this study has demonstrated that the total N loss to the environment can be minimized from combination of manure with chemical fertilizer in rain-fed cropping systems. The estimate of total N loss to the environment was achieved by including soil N status changes in the root zone after long-term fertilization in the mass balance equation. In the highly weathered acidic red soil in south region of China, all fertilization treatments increased the N content as well as downward movement in soil profile suggesting a higher leaching risk. At locations with alkaline soils in the north and central regions, fertilization treatments increased soil N storage mainly in the top soil (0–40 cm depth) indicating a relatively low leaching potential. At all study sites, manure application resulted in a lower total N loss to the environment (ave. ≤24% vs. >35% from all other chemical fertilization treatments). We conclude that manure incorporation in N fertilization can be one of the most effective strategies that not only improve soil fertility/productivity for high crop yield but also significantly reduce the total N loss or environmental impact.

## Methods

### Experimental location, soil property, and climate

The three long-term experiment sites representing three different major agricultural regions were located at Urumqi (Xinjiang province, northwest), Zhengzhou (Henan province, central), and Qiyang (Hunan province, south) of China. Soil types, chemical properties of the soils collected in 1990, and climate conditions for the study locations are given in Table 4. Urumqi represents a typical arid mountain-oasis ecosystem, with a high temperature in the summer and low temperature in the winter. Zhengzhou and Qiyang have a field ecosystem with typical continental monsoon climate and subtropical monsoon climate, respectively. The soil at Qiyang site, developed from Quaternary red clay, had a much lower pH (5.7) than that at Urumqi (8.1) and Zhengzhou (8.3).The soil texture was loamy clay at Qiyang and loam at Urumqi and Zhengzhou.

### Cropping system

A 3 year mono-cropping rotation system with spring wheat-corn-winter wheat was used at the Urumqi site due to the shorter growing season compared to other two sites (Table 5). Corn was sown in early May and harvested in early October in the first year; spring wheat was seeded in mid-April in the second year and harvested in late July; then winter wheat was seeded in late September in the second year and harvested in mid-July in the third year at Urumqi. An annual double cropping system with summer corn-winter wheat rotation was used at both Zhengzhou and Qiyang. Corn was sown in early June and harvested in mid-September; winter wheat was sown in early October and harvested in early June the following year. The cropping systems in all three study sites were rain-fed.

### Experimental design and fertilization management

The same eight treatments tested at each of the three study sites were: unfertilized CK, N, NP, NK, NPK, NPKM, 1.5NPKM, and NPKS for which chemical N, P, and K were applied as urea, Ca_3_(PO_4_)_2_, and KCl, respectively. The manure was from cattle, horses, or pigs depending on local availability and was applied before seeding for all sites. For NPKS treatment at Qiyang and Zhengzhou, only corn stover was returned after fall harvest and wheat straw was removed from the field because of the limited time between wheat harvest and sowing of corn seeds. At Urumqi, both corn stover and wheat straw were returned after annual harvest (NPKS) and we will refer to the straw return for the NPKS treatment in the paper. Half of the N was applied as basal fertilizers prior to seeding and the remaining N fertilizer was applied as a top dressing during the growing season. Both the chemical fertilizers and the manure were applied by banding at a depth of 10 cm, followed by sowing of each crop and then covering with surface soil. Field information including experimental design is described in Duan *et al*.^11^. To obtain a large representative experimental plot, each treatment was not replicate at each individual site but considered replicated at the three different sites. At each site, treatments were completely randomized for first year but the same treatment was applied to the same plot in the following years.

The target fertilizer application rates for high yield were slightly different among the three sites based on local growers’ common practices for the cropping system, cultivars used, climate, and soil conditions. The annual application rate of N was 241.5 kg ha^−1^ for the mono-cropping system at Urumqi, and 353 and 300 kg ha^−1^ for the double cropping systems at Zhengzhou and Qiyang, respectively (Table 5). The total amount of N applied was the same for all fertilization treatments (except for the 1.5NPKM treatment) at each site. For the NPKM treatment, 30% of the total N applied was urea and the remainder was in the form of composted manure. The annual manure application rates varied a little depending on its total N content, which was determined each year. The nutrient contents in manure were listed in Table 5. For the 1.5NPKM treatment, the total N application rate from both the urea and manure was 1.5 times that of the NPKM treatment at all three sites. For the NPKS treatment, corn stover or wheat straw were chopped and incorporated into the soil following harvest. Nutrients (NPK) in the straw or stover, however, were not counted in N applications because of their unknown availability over time.

Depending on the field availability, the treatment plot size was 468 m^2^ (36 m × 13 m), 400 m^2^ (25 m × 16 m), and 200 m^2^ (20 m × 10 m) at the Urumqi, Zhengzhou, and Qiyang sites, respectively. To avoid edge effects, the plots were isolated by 100 cm deep concrete barriers that separated the treatments at each site.

### Soil and plant sampling and analysis

Surface soil (0–25 cm) samples were collected each year, approximately 15 days after the crop harvest in the fall and the sampling method is described in detail in Duan *et al*.^11^. Additionally, soil from the upper most 100 cm was sampled in autumn of 1990 (at the beginning of the experiment) and 2009 (after 19 years of fertilization treatment) to determine changes in soil N storage. All samples were analyzed for total soil N using Kjeldahl method^12^. Twenty undisturbed soil cores were collected from each plot at the depth of 0–20, 20–40, 40–60, 60–80, and 80–100 cm. For each sampling depth, 4 cores from the same plot were randomly chosen and composited for a total of five samples for measurement. Bulk density (BD) was measured using the method described by Culley^28^ and used to calculate the N content (storage) in the soil. Methods for plant sampling and N measurement were detailed in Duan *et al*.^11^.

### Estimation of total N loss to the environment from fertilization

Nitrogen balance (N input - N output by crop harvest removal) has been used as an indicator for potential N loss^29^. The N balance, however, reflects more on crop use efficiency rather than the loss to the environment because changes in soil N status or storage (representing soil fertility or productivity) are not considered in the equation. Our research was to fill this gap because the data on soil N status were determined in our long-term experiment. By including this component into the N mass balance equation, we were able to closely estimate the total N loss to the environment, which is the sum of runoff, leaching, ammonia volatilization, denitrification, and other forms of gas emissions. In all three study sites, runoff and denitrification were considered minimal because of the flat fields and dry-land production environment. The total N loss to the environment would mainly include leaching, ammonium volatilization, and other forms of gas emissions such as nitrous oxide (N_2_O) etc. For corn and wheat, we consider the top 100 cm soil as the rooting zone, where most of the N is considered available for crop uptake, and that below 100 cm depth would be easily subject to leaching loss^30^. Specifically, the N loss was estimated based on soil N content changes after 19 years of fertilization treatments in this study and for easy comparisons with other and/or future studies we converted the loss to annual basis. The following equations were used to estimate the annual N loss to the environment:


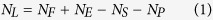


where N_L_, N_F_, N_E_, N_S_, and N_P_ are the N loss to the environment, application rate from the fertilizer and manure, supplied from the environment, changes in soil, and plant uptake, respectively. All units are in kg N ha^−1^ yr^−1^.

The N_E_ via soil N mineralization, N fixation, and precipitation was estimated from the sum of soil N change and plant uptake in the control (CK) where no N fertilizer was applied. The N_P_ was measured and reported previously in Duan *et al*.^12^. The N_S_ was calculated as:


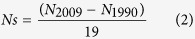


where N_2009_ and N_1990_ are the N contents (kg ha^−1^) in soil profile at 2009 and 1990, respectively.


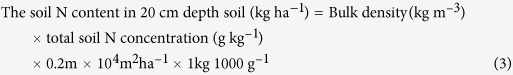


The following equations were used to evaluate the fate of the N applied during the 19 years of fertilizer applications in this paper:


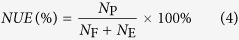



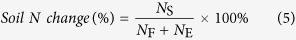



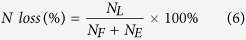


### Statistical analyses

Nitrogen distribution in profile soil can partially reflect its downward movement to signal leaching risks. Due to lack of replicates at each individual site, we considered that the treatments were replicated at the three different sites. For soil N content in the profile, a mixed model was used to statistically analyze the effects of treatments and depth, as well as their interactions. In the model, the treatments and depths were set as fixed effects and the site was set as a random effect. The Student-Newman-Keuls method was used for multiple comparisons to determine significant differences among the means of the soil profile N. Similarly for the fate of N data, a two-way ANOVA and a mixed model with both fixed and random effects where treatments were fixed effects and the sites were random effects. Multiple comparisons for treatment effects on the percentage of soil N increased, N uptake by crops, and N loss to the environment for data from all three sites were performed using the Turkey-Kramer method to avoid Type I error. All analyses were conducted using SAS 9.2 (SAS Institute, Cary, NC, USA).

## Additional Information

**How to cite this article**: Duan, Y. *et al*. Long-term incorporation of manure with chemical fertilizers reduced total nitrogen loss in rain-fed cropping systems. *Sci. Rep.* **6**, 33611; doi: 10.1038/srep33611 (2016).

## Figures and Tables

**Table 1:** Tests of fixed effects on soil profile N content in the long-term (1990–2009) fertilization experiment.

Effect	Num. DF	Mean Square	F Value	Pr > F
Depth	4	7.94	37.12	<0.0001
Treatment	7	1.21	5.77	<0.0001
Depth × Treatment	28	0.30	1.40	0.096

**Table 2:** Statistical analysis on differences in soil profile N content (Mg ha^−1^) among the fertilization treatments from all three study sites in the long-term experiment (1990–2009).

Treatment	CK	N	NP	NK	NPK	NPKM	1.5NPKM	NPKS
Mean^*^ (Mg N ha^−1^)	7.3 ± 0.5 c^*^*	8.4 ± 0.9 bc	8.8 ± 0.7 bc	8.4 ± 1.0 bc	8.0 ± 0.9 bc	9.1 ± 0.6 b	10.2 ± 0.5 a	8.6 ± 0.5 bc
Differences between soil depths								
Depth (cm)	0–20	20–40	40–60	60–80	80–100			
Mean^*^ (Mg N ha^−1^)	2.6 ± 0.2 a	2.0 ± 0.2 b	1.4 ± 0.2 c	1.4 ± 0.1 c	1.3 ± 0.1 c			

^*^Mean ± standard deviation. ^**^Different letters in the same row indicate significant differences between treatments or depths at *P* < 0.05 according to Student-Newman-Keuls method. The abbreviations of fertilization treatments are the same as presented in Fig. 1.

**Table 3:** The fate of N applied after long term (1990–2009) applications of chemical fertilizers and manure/stover treatments at Urumqi, Zhengzhou and Qiyang.

Site	Treatment	N_F_ (kgha^−1^yr^−1^)	N_S_ (kg ha^−1^yr^−1^)	Soil N change (%)	N_P_ (kg ha^−1^yr^−1^)	NUE (%)	N_L_ (kg ha^−1^yr^−1^)	N loss (%)
Urumqi	CK	0	−27		43		0	
	N	241.5	−4	−2	86	33	175.5	68
	NP	241.5	6	2	126	49	125.5	49
	NK	241.5	−31	−12	84	33	204.5	79
	NPK	241.5	−35	−14	134	52	158.5	62
	NPKM	241.5	67	26	139	54	51.5	20
	1.5NPKM	362.2	164	43	143	38	71.2	19
	NPKS	241.5	10	4	113	44	134.5	52
Zhengzhou	CK	0	−43		83		0	
	N	353	−8	−2	127	32	274	70
	NP	353	36	9	284	72	73	19
	NK	353	−3	−1	155	39	241	61
	NPK	353	12	3	300	76	81	21
	NPKM	353	53	13	276	70	64	16
	1.5NPKM	529.5	91	16	326	57	152.5	27
	NPKS	353	38	10	289	74	66	17
Qiyang	CK	0	−8		20		0	
	N	300	109	35	43	14	160	51
	NP	300	112	36	80	26	120	38
	NK	300	127	41	55	18	130	42
	NPK	300	58	19	104	33	150	48
	NPKM	300	84	27	153	49	75	24
	1.5NPKM	450	130	28	205	44	127	27
	NPKS	300	77	25	122	39	113	36
Average	CK	0	−26		49		0	
	N	298	32	10 cd^*^	85	27 b	203	63 a
	NP	298	51	16 bc	163	49 ab	106	35 ab
	NK	298	31	9 cd	98	30 b	192	61 a
	NPK	298	12	3 d	179	54 ab	130	43 ab
	NPKM	298	68	22 ab	189	58 a	64	20 b
	1.5NPKM	447	128	29 a	225	47 ab	117	24 b
	NPKS	298	42	13 bc	175	52 ab	105	35 ab

N_F_: N application rate from the fertilizer and manure; N_S_: N change in soil; N_P_: Plant uptake of N; N_L_: N loss to the environment; NUE: nitrogen use efficiency.

*Different letters in the same column indicate significant differences between treatments (*P* < 0.05). The abbreviations of fertilization treatments are the same as presented in Fig. 1.

**Table 4:** Geographic, climate and soil conditions (0–25 cm) of the three long-term experimental sites in China.

Parameter\Location name	Urumqi (Northwest)	Zhengzhou (Central)	Qiyang (South)
Geographic and climate conditions			
Altitude (m)	600	59	120
Latitude	43°95′26″	34°47′25″	26°45′12″
Longitude	87°46′45″	113°40′42″	111°52′32″
Mean annual temperature (°C)	7.7	14.0	18.0
Annual precipitation (mm)	310	632	1250
Annual evaporation (mm)	2570	1450	1470
Annual mean frost-free days	156	224	300
Soil conditions*			
Soil classification in FAO	Haplic Calcisol	Calcaric Cambisol	Eutric Cambisol
Texture type	Loamy clay	Light loam	Light loam
Clay (<0.002 mm) (%)	20.9	13.4	40.9
Soil pH (water extraction, 1:2.5 w/v)	8.1	8.3	5.7
Organic carbon (Mg ha^−1^)	22	20	14
CaCO_3_ content (Mg ha^−1^)	162	144	0
Total (Mg ha^−1^)			
N	2.2	3.0	2.5
P	1.7	1.9	1.1
K	58	50	33
Available (kg ha^−1^)			
Alkaline hydrolysable N	138	228	188
Olsen-P	9	19	26
NH_4_OAc-K	720	221	290

^*^Soil parameters were measurements for soil samples collected before the field experiment establishment in 1990.

**Table 5:** Annual application rate (kg ha^−1^ yr^−1^) of chemical fertilizers and manure for various treatments during the long-term (1990–2009) experiment at three study sites.

Experimental site	Urumqi	Zhengzhou	Qiyang
Crop rotation	Spring wheat-corn- winter wheat (3 yr)	Corn- wheat (1 yr)	Corn- wheat(1 yr)
Added N(kg ha^−1^yr^−1^)	241.5	330–353*	300
Added P (kg ha^−1^yr^−1^)	50.2	72–77*	52.4
Added K (kg ha^−1^yr^−1^)	50.4	137.5–146.5*	99.7
Manure type	Cow	Horse or cow	Pig
Nutrient content in manure (g kg^−1^)			
N	2.1–4.6	6.3–21.4	3.7–8.7
P	0.8–1.1	1.0–4.6	1.2–3.5
K	1.7–2.6	2.3–10.3	1.8–6.2

^*^The lower application rate was used in 1991 and adjusted to the higher rate from 1992 to 2009 at the Zhengzhou experimental site.
